# Characteristics of Human Amniotic Fluid Mesenchymal Stem Cells and Their Tropism to Human Ovarian Cancer

**DOI:** 10.1371/journal.pone.0123350

**Published:** 2015-04-16

**Authors:** Liru Li, Dejun Wang, Jun Zhou, Yan Cheng, Tian Liang, Guangmei Zhang

**Affiliations:** Department of Genecology and Obstetrics, Harbin Medical University, Harbin, Heilongjiang, China; University of Toyama Graduate School of Medicine and Pharmaceutical Sciences, JAPAN

## Abstract

The mesenchymal stem cells (MSCs) derived from amniotic fluid (AF) have become an attractive stem cells source for cell-based therapy because they can be harvested at low cost and avoid ethical disputes. In human research, stem cells derived from AF gradually became a hot research direction for disease treatment, specifically for their plasticity, their reduced immunogenicity and their tumor tropism regardless of the tumor size, location and source. Our work aimed to obtain and characterize human amniotic fluid mesenchymal stem cells (AFMSCs) and detect their ovarian cancer tropsim in nude mice model. Ten milliliters of twenty independent amniotic fluid samples were collected from 16-20 week pregnant women who underwent amniocentesis for fetal genetic determination in routine prenatal diagnosis in the first affiliated hospital of Harbin medical university. We successfully isolated the AFMSCs from thirteen of twenty amniotic fluid samples. AFMSCs presented a fibroblastic-like morphology during the culture. Flow cytometry analyses showed that the cells were positive for specific stem cell markers CD73,CD90, CD105, CD166 and HLA-ABC (MHC class I), but negative for CD 45,CD40, CD34, CD14 and HLA-DR (MHC class II). RT-PCR results showed that the AFMSCs expressed stem cell marker OCT4. AFMSCs could differentiate into bone cells, fat cells and chondrocytes under certain conditions. AFMSCs had the high motility to migrate to ovarian cancer site but didn’t have the tumorigenicity. This study enhances the possibility of AFMSCs as drug carrier in human cell-based therapy. Meanwhile, the research emphasis in the future can also put in targeting therapy of ovarian cancer.

## Introduction

Mesenchymal stem cells (MSCs) are adult non-hematopoietic stem cells, which widely exist in the matrix of various organs and tissues. They have a high degree of proliferation, self-renewal and multi-directional differentiation ability. In recent years, the research progress of MSCs develops quickly due to the improvement of the MSCs’ culture conditions and detection means. MSCs can be extracted from many sources such as bone marrow (BM), adipose tissue, umbilical cord, peripheral blood and placenta [1–4]. The cells are considered useful delivery vehicles for many types of solid tumors [5–11] as they can migrate to tumor lesions [12] regardless of the tumor size, location and source. In the past, the human bone marrow-derived mesenchymal stem cells were the most widely researched, but their application was limited as they could only be obtained through bone marrow biopsy. In 2003, Prusa etc. [13] found that amniotic fluid contained abundant stem cells. Amniotic fluid stem cells showed the characteristics of MSCs with the ability of highly proliferation, self-renewal and multiple differentiations potential. As the amniotic fluid can be gained through the amniotic cavity puncture and the operation has little impact on fetal and maternal, it is not only with high security, but also avoids the ethical issues related to embryonic stem cells [14–16], so the amniotic fluid mesenchymal stem cells get high attention of scientists [17]. Recent studies showed that AFMSCs had gradually become the hot spot in the research of immune regulation, repair medicine and tumor targeted therapy.

In the past 30 years, with the development of surgery and new drugs (such as paclitaxel, docetaxel, Gemcitabine), the survival of ovarian cancer had a great progress, but the ovarian cancer now is still the greatest killer in the gynecology malignant tumor. At present the treatments of ovarian cancer include surgery, chemotherapy, radiotherapy and biological therapy. Biological treatment is the fourth-largest mode of combination therapy for tumor, and it is approved by more and more patients and their families. Targeted therapy of tumor is a blend of the multidisciplinary technology of new medicine, and it is an important research direction. MSCs based targeted drug delivery system has a distinct advantage, namely it has the unique characteristics of eosinophilic tumor lesion site, and compared with other targeted drug delivery system, MSCs can migrate to tumor lesions [12] regardless of the tumor size, location and source. Due to tumor tropism of mesenchymal stem cells, we aimed to study the ovarian cancer tendency of amniotic fluid mesenchymal stem cells, which may provide new ideas for the targeted therapy of ovarian cancer[18–19].

From what have been described above, We propose to investigate the characteristics of amniotic fluid mesenchymal stem cells and their tropism to ovarian cancer, laying the groundwork for ovarian cancer targeted therapy[20–21].

## Materials and Methods

### Speciments source

Ten milliliters of twenty independent amniotic fluid samples were collected from 16–20 week pregnant women who underwent amniocentesis for fetal genetic determination in routine prenatal diagnosis in the first affiliated hospital of Harbin medical university. The patients signed informed consent before the participation. This study got the approval of Harbin medical university ethics committee. All animal experiments were carried out in Balb/c nude mice, aged approximately 6 weeks, weighing 17–19g, obtained from slack company in Shanghai and animal experiments were carried out in strict accordance with the recommendations in the Guide for the Care and Use of Laboratory Animals of the Harbin medical university ethics committee. The protocol was approved by the Committee on the Ethics of Animal Experiments of the Harbin Medical University. All efforts were made to minimize suffering. Human embryonic stem cells (HESCs) were bought from American ATCC. SKOV3 cells and human skin fibroblast cells (HSFCs) were independently preserved by the obstetrics and gynecology laboratory in the first affiliated hospital of Harbin medical university, all cells operation got the approval of Harbin medical university ethics committee.

### Reagents source

Alpha MEM Medium, RPMI—1640 Medium, DMEM/High Glucose Medium, Fetal Bovine Serum FBS, Penicillin-Streptomycin, Trypan Blue, TRYPSIN, EDTA, Trizol, Fluorescent tags mouse anti human antibody: Mouse IgG1-PE, Mouse IgG1-FITC, CD90—FITC, CD105—FITC, CD73—FITC, CD40—FITC, CD45—FITC and CD34—FITC, CD14—PE, HLA—ABC—FITC, HLA—DR-PE, PCR Primers, One-step RT-PCR kit, Cell Calcium Alizarin Red Staining Kits, Oil red O, Alcian Blue, Human Mesenchymal Stem Cells Chondrogenic Differentiation Medium, 1,1′-Dioctadecyl-3,3,3′,3′-tetramethylindocarbocyanine perchlorate-Dil, Primary antibody mouse anti-human CD90, secondary antibody goat anti-mouse, DAPI.

### Main mediums

Growth medium for AFMSCs:89%α-MEM, 10%FBS, 1%Penicillin-Streptomycin

Growth medium for HESCs:89%α-MEM, 10%FBS, 1%Penicillin-Streptomycin

Growth medium for SKOV3 cells:89%RPMI—1640 Medium, 10%FBS, 1%Penicillin-Streptomycin

Growth medium for HSF cells:89%DMEM/High Glucose, 10%FBS, 1%Penicillin-Streptomycin

Osteogenic Differentiation Medium consisted of 89%alpha MEM, 10% FBS, 1% Penicillin-Streptomycin, 0.1 u mol/L dexamethasone, 10mmol/L beta glycerin sodium phosphate and 50 u mol/L ascorbic acid.

Adipocyte Differentiation Medium consisted of 89%alpha MEM, 10% FBS, 1% Penicillin-Streptomycin, 60 ul/mL indomethacin, 0.5mmol/L 3—isobutyl methyl xanthine, 1 umol/L dexamethasone and 5 u g/L insulin.

### AFMSCs isolation and culture

10 mL of AF was extracted under aseptic conditions and transported to the laboratory within 2h in an ice box. Each sample was centrifuged at 1,000 rpm for 10 mins [22]. After Trypan Blue staining, the cell precipitation was plated at an initial density of 1×10^5^ cells/cm2 in a 25 cm2 tissue culture treated flask in alpha modified Eagle’s medium (a-MEM) supplemented with 10% (vol/vol) fetal bovine serum (FBS) 1% Penicillin-Streptomycin and 5ng/ml basic fibroblast growth factor, and incubated for 7 days in a humidified 37°C incubator under 5% CO_2_ until the first colonies appeared. The spindle-shaped AF-MSC colonies were selected and subcultured. Subsequently cultures were washed with PBS and cell in liquid every 3–4 days until the cell population reached 70–80% confluence, and the cells were trypsinized (0.25% trypsin and 0.01% EDTA; w/v) to single cell from the plate. Subcultured cells were batched onto new plates with a ratio of 1:2 and marked as passage 1(P1). The cells during third to fifth passages were used for experiments.

### Characterization of AFMSCs

#### Morphological Observation of AFMSCs

Morphology observation adopted inverted phase contrast microscope. Observing the growthform of the AFMSCs in different passages.

#### AFMSCs growth curve and population doubling time PDT

Well growing cells from passages3,5,10,15,20 were harvested by trypsinization(0.25% trypsin and 0.01% EDTA; w/v), resuspended in a-MEM containing 10% (vol/vol) FBS at a concentration of 1×10^4^ cells/ml, plated into 24-well plates at 5000 cells (500ul) per well, and incubated 24 hours in a humidified 37°C incubator under 5% CO2 to allow the cells to adhere to the plates. The cells from three random wells were counted each day for 8 days and the mean values were used to plot cells growth curves. The population doubling time was calculated according to the following formula:

DT=[lg2/lgNt−lgNo]×t

where *Nt* = ultimate cell number; *No* = primary cell number; *t* = termination incubation time.

#### Stem cells specific marker OCT 4 expressing in AFMSCs

Reverse transcription-polymerase chain reaction (RT-PCR) was used for the detection of OCT4 expression in AFMSCs from passages 3, 5, 10, 15 and 20. Human embryonic stem cells were used as positive control and human skin fibroblast cells were used as negative control. For preparing of RNA for reverse transcription-polymerasechain reaction (RT-PCR), total RNA was extracted using Trizol according to the manufacturers’ protocol. RNA concentrations were measured by absorbance at 260 nm with a spectrophotometer and 1 μg RNA treated by DNase I of the sample was used as a template for a one-step RT-PCR system. The RT-PCR was carried out using the following cycling conditions: 94°C for 15 min; then 35 cycles of 94°C for 30s, 57°C for 30s, and 72°C for 30s; and finally 72°C for 10 min. PCR products were visualized on a 1% agarose gel with ethidium bromide and detected under a UV transilluminator. Primer sequences used in the study are listed in Table 1.

**Table 1:** Primer sequences used in the study.

Gene	Primers	Products(bp)
4-Oct	F:5’-CGTGAAGCTGGAGAAGGAGAAGCTG-3’	247
	R:5’-CAAGGGCCGCAGCTTACACATGTTC-3’	
β-actin	F:5’-TGGCACCACACCTTCTACAATGAGC-3’	396
	R:5’-GCACAGCTTCTCCTTAATGTCACGC-3’	
RUNX2	F:5’-CGCAAAACCACAGAACCACAAGTGCG-3’	164
	R:5’-GTTGGTCTCGGCTGGTAG-3’	
PLIN	F:5’-AAACAGCATCAGCGTTCCCATC-3’	173
	R:5’-AGTGTTGGCAGCAAATTCCG-3’	
ACAN	F:5’-CGGGTCTCACTGCCCAACTACCCG-3’	200
	R:5’-GCCTTTCACCACGACTTCCAG-3’	

### Immunophenotypes of AFMSCs

Flow cytometric analysis was performed to detect the phenotypes of AFMSCs including CD166, CD105, CD90, CD73, CD45, CD34, CD14, CD40, HLA-ABC and HLA-DR[21]. Well growing cells from passages 3 to 5 were harvested by trypsinization(0.25% trypsin and 0.01% EDTA; w/v), resuspended in PBS at a concentration of 2×10^4^ cells/20ul. Then cells were stained for 20 minutes at room temperature according to the antibody instructions. Stained cells were resuspended in 300ul PBS, analysed using BD FACSCalibur flow cytometer. The computed data were analysed using CellQuestPro software provided by the manufacturer.

### AFMSCs differention in vitro

The 1×10^5^ AFMSCs at the third to fifth passages were treated with Osteogenic Differentiation Medium for 2 weeks. Cells cultured in alpha-MEM growth medium were taken as the control group. Cells were replaced fresh medium every three days. After 2 weeks, osteogenesis was assessed by alizarin red staining and RT-PCR detecting the expression of Runt related transcription factor 2 RUNX2.

The 1×10^5^ AFMSCs at the third to fifth passages were treated with Adipocyte Differentiation Medium for 2 weeks. Cells cultured in alpha-MEM growth medium were taken as the control group. Cells were replaced fresh medium every three days. After 2 weeks, adipocyte was assessed by Oil-red O staining and RT-PCR detecting the expression of perilipin PLIN.

The 1×10^5^ AFMSCs at the third to fifth passages were treated with Chondrogenic Differentiation Medium for 3weeks. Cells cultured in alpha-MEM growth medium were taken as the control group. Cells were replaced fresh medium every three days. After 3 weeks, chondrogenic was assessed by Alcian Blue staining and RT-PCR detecting the expression of aggrecan ACAN.

### AFMSCs and SKOV3 tumorigenicity analysis

Mice were kept on a 14-h light/10-h dark cycle and provided with sterility food and water and the mice were allowed to acclimate to SPF condition for 7 days prior to inclusion in the tumorigenicity experiments. Mice were randomly separated into 2 groups(n = 6 per group): AFMSCs and SKOV3 tumorigenicity groups. AFMSCs tumorigenicity models of nude mice were established by injecting 0.2ml physiological saline with 6×10^6^AFMSCs per milliliter in right scapular subcutaneous and the contralateral side were injected with 0.2ml physiological saline as control. SKOV3 tumorigenicity models of nude mice were established by injecting 0.2ml physiological saline with 6×10^6^ SKOV3 cells per milliliter in right scapular subcutaneous and the contralateral side were injected with 0.2ml physiological saline as control. Tumor formation situation in two groups was observed every day. After 60 days, those 12 mice were killed by the dislocation, and both left and right scapular subcutaneous tissues were collected. By macroscopic observation and HE staining to observe if there were any tumor formation, then assayed the tumorigenicity of AFMSCs and SKOV3 cells.

### Tropism of AFMSCs to human ovarian cancer

Mice were kept on a 14-h light/10-h dark cycle and provided with sterility food and water and the mice were allowed to acclimate to SPF condition for 7 days prior to inclusion in the following experiments. For the establishment of ovarian cancer, 6×10^6^ SKOV3 cells were administered subcutaneously in 0.2mL of physiological saline into the right scapular of each nude mouse(n = 12). One week later, the nude mice ovarian cancer models were established. 12 nude mice were randomly divided into two groups(n = 6 per group): experimental group and control group. Labeling the AFMSCs with Dil prior to injection. In the experimental group (n = 6) 6×10^6^ AFMSCs suspended in saline were intravenously (i.v.) administered into the tail vein of the mouse in a volume of 0.2ml and the mice in control group (n = 6)were injected with 0.2ml physiological saline. The two groups injections were weekly performed. One week after the third injections, animals were killed by the dislocation, and tumors, kidneys, spleens lungs and livers of two groups were collected to evaluate the expression of CD90 in these tissues by immunohistochemical and detect the red fluorescence expression in these tissues by the laser confocal after stained by DAPI.

## Results

### Isolation, culture and morphological Observation of AFMSCs

**Fig 1:**
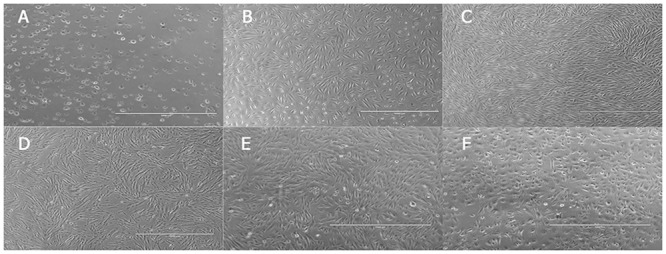
Morphology of different passages of AFMSCs (Scale bar = 1000um). (A)The non adherent cells; (B) The primary passage; (C) The fifth passage;(D) The tenth passage;(E)The fifteenth passage; (F) The twentieth passage.

AFMSCs were successfully isolated through the centrifugal adherent method from thirteen of twenty amniotic fluid samples. Primary stem cells collected from the second trimester of pregnancy amniotic fluid adhered to plates at 5–7 days later and presented short polygon or fusiform. About 10 days later, part of the cells turned globular or strip. The cell body assumed spindle. With the rapid proliferation of cells, we observed more cell clones appeared. Three weeks later the cells reached 80%-90% confluency. At this time these cells presented relatively uniform long spindle which were similar to bone marrow mesenchymal stem cells. With the increase of cell passage, the shape of cells became slender. Cells could extend continuously to 20 generations and after the 20th generation cell morphological began to change, cell body increased, arranged loosely and cell edge turned burr shaped.(Fig 1)

### AFMSCs growth curve assay

**Fig 2:**
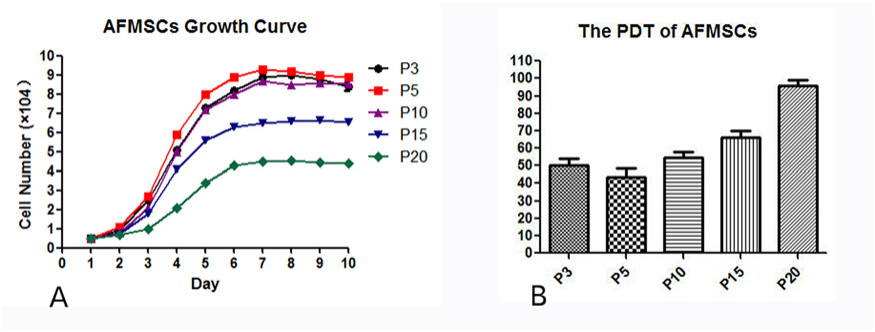
The growth curve and PDT of AFMSCs in passage 3,5,10,15 and 20. (A) The growth curve of AFMSCs in different passages. (B) The PDT of AFMSCs in different passages.

The cell growth curves at P3,P5,P10,P15 and P20 were shown in Fig 2-1. The growth curves were roughly similar to shape “S”. The cells stayed in detention period after passaging 2 days, then turned to logarithmic phase and seven days later the cell number no longer increased. The proliferation of cells in tenth generation was slower than in third and fifth generation. The PDT (population double time) of AFMSCs was shown in Fig 2-2. It was determined to be 50, 48, 55,67and 97 h for P3, P5, P10,P15 and P20, respectively.

### Stem specific marker OCT4 expressing in AFMSCs

**Fig 3:**
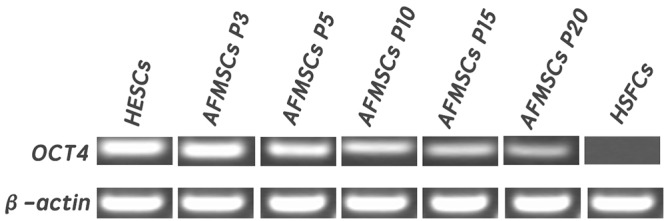
Stem cells specific marker Oct—4 expressing in AFMSCs in passage 3,5,10,15 and 20. Human embryonic stem cells were used as positive control and human skin fibroblast cells were used as negative control. β-action served as the internal control. Oct4 expressed positively in AFMSCs and the expression of Oct4 gradually decreased as the passage number increased.

In this part, human embryonic stem cells were used as positive control and human skin fibroblast cells were used as negative control. The comprehensive analysis of RT-PCR showed that OCT4 expressed positively in AFMSCs and the expression of OCT4 gradually decreased as the passage number increased(Fig 3).

### Immunophenotypes of AFMSCs

**Fig 4:**
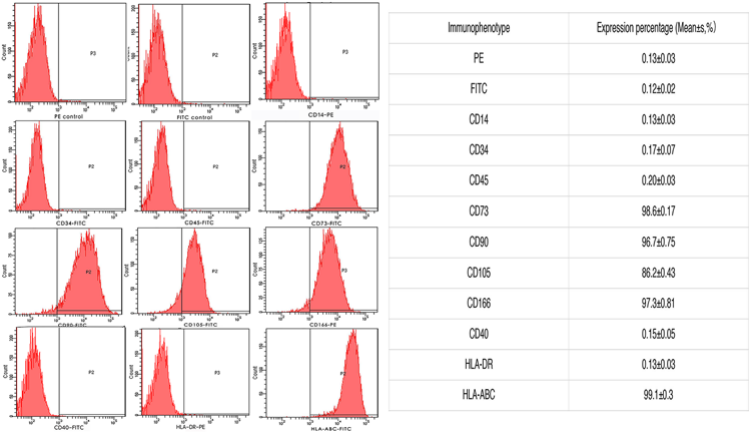
Immunophenotypes of AFMSCs. Flow cytometry analysis showed that the AFMSCs positively expressed CD73 CD90, CD105 and CD166, but negatively expressed CD34, CD45 and CD14. The results also showed that the AFMSCs expressed HLA-ABC(MHC class I), but not HLA-DR(MHC class II) nor CD40.

Based on Flow cytometry analysis, we found that the AFMSCs were positive for CD166, CD90,CD73 and CD105, but negative for CD34, CD45 and CD14, representing characteristic phenotypes of mesenchymal stem cells. The results also showed that the AFMSCs expressed HLA-ABC(MHC class I), but not HLA-DR(MHC class II), nor CD40, manifesting the low immunity of AFMSCs (Fig 4).

### AFMSCs differention in vitro

**Fig 5:**
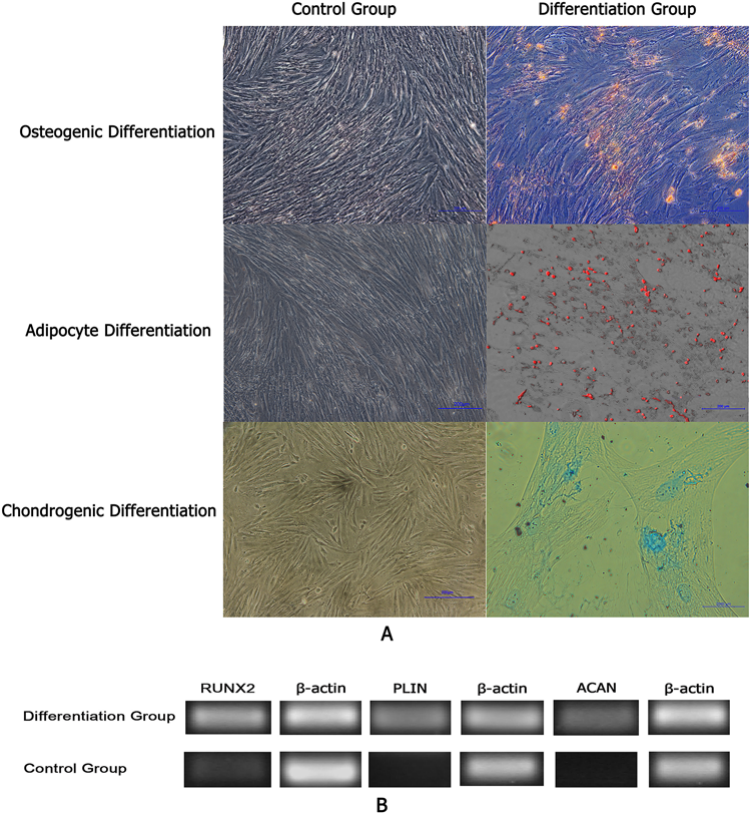
AFMSCs differention in vitro. Fig 5A The staining results of AFMSCs after being cultured in different mediums Fig 5B RT-PCR results of AFMSCs in different mediums.

After being cultured in complete medium for two weeks, the negative control cells were not stained by Alizarin Red staining (Fig 5A) and RUNX2 expressed negatively (Fig 5B). AFMSCs put up obviously appearance changes after incubated in osteogenic induction medium for 14 days and cells were capable of osteogenic differentiation as they were positive by Alizarin Red staining (Fig 5A) and RT-PCR showed positive expression of RUNX2 (Fig 5B).

After being cultured in complete medium for two weeks, the negative control cells were not stained by Oil Red O staining (Fig 5A) and PLIN expressed negatively (Fig 5B). With staining by Oil Red O, AFMSCs could differentiate into adipogenic cells after 14 days post-induction (Fig 5A). Meanwhile, the expression of PLIN gene presented positive (Fig 5B).

After being cultured in complete medium for 21 days, the negative control cells were not stained by Alcian blue staining (Fig 5A) and ACAN expressed negatively (Fig 5B). 21 days after being cultured in the chondrogenic differentiation medium, AFMSCs showed positive staining of Alcian blue (Fig 5A) and expressed chondrocyte specific gene ACAN (Fig 5B). It indicated that AFMSCs could differentiate into cartilage cells in specific conditions.

### AFMSCs and SKOV3 cells’ tumorigenicity analysis

**Fig 6:**
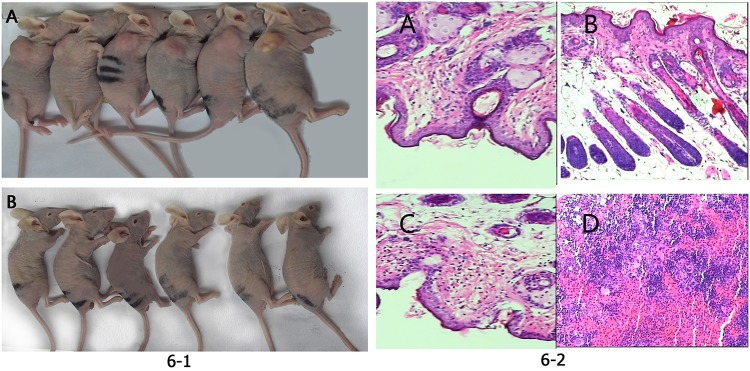
AFMSCs and SKOV3 cells tumorigenicity analysis by macroscopic and HE staining observation. Fig 6–1 Macroscopic observation.(A) SKOV3 cells formatted tumors on the right side of the 6 nude mice.(B) No tumor were seen in the right side of the the 6 AFMSCs injection nude mice. Fig 6–2 Nude mice scapular subcutaneous tissues HE staining. (A) Saline injection site HE staining in AFMSCs injection group.(B) AFMSCs injection site HE staining in AFMSCs injection group.(C)Saline injection site HE staining in SKOV3 cells injection group.(D) SKOV3 cells injection site accumulated many inflammatory cells.

Two days after the AFMSCs injection, the right side of the nude mice in the AF injection group showed soft skin rashes at the injection site and no skin rashes formatted on the left site.Three days later, the skin rashes on both sides of the 6 nude mice disappeared. 60 days after the injection, the mice were sacrificed, both the AFMSCs injection and saline injection scapular subcutaneous tissues were collected. By macroscopic observation (Fig 6-1)and HE staining(Fig 6-2), we found that no tumor generated. It meaned that the AFMSCs had no tumorigenicity and might be used in vivo safely.

Two days after the SKOV3 cells injection, the right side of the 6 nude mice showed soft skin rashes at the injection site and no skin rashes appeared on the left site.Three days later, the skin rashes on the right side of the nude mice still existed, while no tumor formatted on the left scapular subcutaneous.7 days after the injection, slightly hard round mass were visible on the right subscapular of the 6 nude mice. And as time went on, the tumor size increased. 60 days after the injection, the mice were sacrificed, both the SKOV3 injection and saline injection scapular subcutaneous tissues were collected. By macroscopic observation (Fig 6-1)and HE staining(Fig 6-2), we found that tumor generated on the right side of the 6 nude mice, while no tumor formatted on the left side. It meaned that the SKOV3 cells had the tumorigenicity. They could be used for the subsequent establishment of ovarian cancer in nude mice model.

### The tropism of AFMSCs to human ovarian cancer

**Fig 7:**
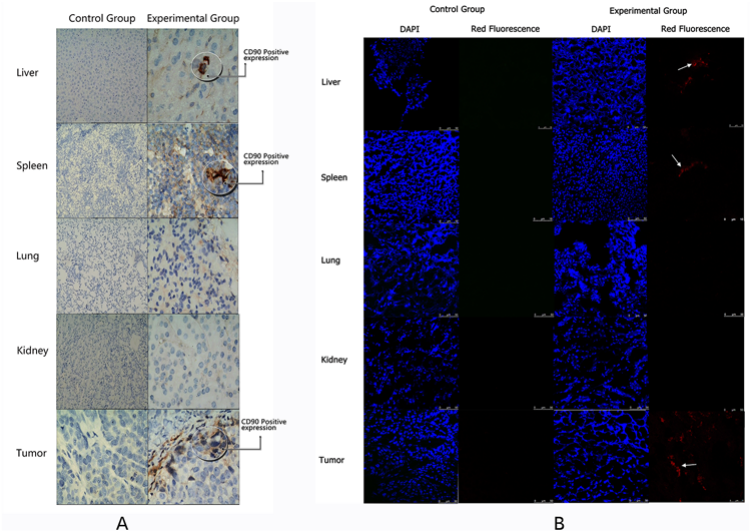
Detecting the tropism of AFMSCs to human ovarian cancer by immunochemistry. (A) The expression of CD90 in liver, spleen, lung, kidney and tumor in both groups. (B) Laser scanning confocal detected the expression of the red fluorescence in liver, spleen, lung, kidney and tumor in both groups.

CD90 immunohistochemical results show that the CD90 mainly expressed in the tumor after the mice were injected with AFMSCs, and only a few AFMSCs were observed in the liver and spleen. The control group showed negative CD90 expression in the tumor and other tissues(Fig 7A) From the laser scanning confocal results we could see the red fluorescent labeled amniotic fluid mesenchymal stem cells in the experimental group with mainly gathered in the tumor site, and small number of stem cells appeared in the liver and spleen. The negative control group showed no red fluorescence(Fig 7B). The results might prompt that the AFMSCs had the tropism to ovarian cancer but not the normal tissue. The exhilarated discoveries also indicated the possibility of ovarian cancer cell-based targeted therapy.

## Discussion

Till now, ovarian cancer is still a very common malignant tumor of female reproductive system around the world. Surgical treatment is not only the first selection of early ovarian cancer treatments but also an indispensable part of the advanced ovarian cancer comprehensive treatment. Although the methods and techniques of surgery and radiation therapy technology is improving, but in quite a long time, we can not improve the 5-year survival rate of ovarian cancer. So to explore a new treatment for ovarian cancer is an urgent matter.

Amniotic fluid mesenchymal stem cells have the characteristics of high proliferative capacity, multi-directional differentiation and extending potential. These indicate that AFMSCs may be used for tissue reconstruction. Besides, as AFMSCs expressed MHCI class molecules HLA—ABC, but didn't express MHC II class molecular HLA—DR nor costimulatory factor CD40, the cells showed low immunogenicity. It can betoken that the amniotic fluid mesenchymal stem cells can be used in vivo experiment without immune rejection.

After being injected with amniotic fluid mesenchymal stem cells, the nude mice showed no tumor formation through macroscopic and HE staining observation, suggesting that amniotic fluid mesenchymal stem cells may be safely used in vivo without causing tumor formation. But the sample of the experiment is still not enough, we need more AF samples to study. Meanwhile, whether amniotic fluid mesenchymal stem cells may cause tumors after being injected into other parts of the mice still needs to study.

The other big advantage of AFMSCs is the tumor tissue tropism. There are a lot of literatures in the world proving that the mesenchymal stem cells have the tropism to tumor tissues, especially to solid tumors[23–26]. This experiment judged whether AFMSCs had the ovarian cancer tropism by injecting the red fluorescent labeled AFMSCs into the tail vein of ovarian cancer mice model, then detecting the expression of CD90 by immunohistochemical and the red fluorescent by laser scanning confocal. We found that AFMSCs mainly gathered in the tumor site and a few cells appeared in liver and spleen. This phenomenon may occur as many studies show that MSCs exhibit tropism for sites of tissue damage and the tumor microenvironment[27–30]. The ovarian tumor may produce some substances such as proinflammatory molecules to prompt AFMSCs tend to the tumor site in the process of tumor formation. A few cells appeared in the liver, spleen could indicate the AFMSCs probably remain in the rich blood supply organs in the process of migrating to the site of the tumor. Besides, tumor formation needs angiogenesis to provide adequate blood nutrient, this could imply that AFMSCs may more easily migrate to rich blood supply parts. The specific mechanism of AFMSCs’ tropism to ovarian cancer also provides a new research direction for us.

In general, with the characteristics of low immunity, no tumorigenicity and ovarian cancer tropism, the AFMSCs may be used as a good carrier of drug for ovarian cancer targeted therapy.
